# Chemotaxonomy compared to morphological and anatomical taxonomy of five *Hibiscus* species

**DOI:** 10.1007/s10265-024-01566-9

**Published:** 2024-08-15

**Authors:** Hala M. E. Abdelfattah, Hussein A. Hussein, Samir S. Teleb, Marwa M. El-Demerdash, Nelly M. George

**Affiliations:** Department of Botany and Microbiology, Faculty of Science, Zagazig University, Zagazig, 44511 Egypt

**Keywords:** Chemotaxonomy, GC–MS, Leaf and petiole anatomy, Leaf epidermal morphology, Phytochemical profiling

## Abstract

*Hibiscus* is a charismatic genus of the Malvaceae family that is noted for its diversity, lacking identifiable characteristics for distinguishing its various species. Therefore, there is an urgent need to develop authentication methods for genus delimitation and species delineation. The present study aims to discern the taxonomic relationships between the well-known, globally familiar, and economically important five *Hibiscus* species, namely: *H.* × *rosa-sinensis*, *H. sabdariffa*,* H. schizopetalus*,* H. syriacus* and *H. tiliaceus* based on traditional morphological and anatomical characteristics compared to the contemporary chemotaxonomy. In this context, the leaf-based methanolic extracts of the studied species were analyzed by Gas Chromatography-Mass Spectrometer (GC–MS) to estimate their secondary metabolites similarity. In addition, selected qualitative morphological and anatomical traits including leaf venation patterns, epidermal micromorphology, stomata types and trichomes diversity, petiole serial sectioning (outline, adaxial groove features, vasculature traces arrangement), and midrib characteristics of the studied species were investigated. The results of both chemotaxonomy and traditional taxonomy exhibited a remarkable agreement in the delineation of the five studied species. Specifically, the chemotaxonomy-based dendrogram separates the studied species into two main clusters with the* H. sabdariffa* as an outlier species in a single cluster and the remaining four species as another cluster with variant distances in its similarity indices. Similarly, the traditional morphological and anatomical characteristics revealed distinct traits for* H. sabdariffa* compared to the remaining four species. The findings of this study highlight the significance of integrating the structural features with phytochemicals profiling as a potential approach that could be harnessed for the delineation of the taxonomically challenging *Hibiscus* genus.

**Supplementary Information:**

The online version contains supplementary material available at 10.1007/s10265-024-01566-9.

## Introduction

*Hibiscus* L. has been delimited as a member of the Malvaceae family, which belongs to the subfamily Malvoideae and tribe Hibisceae (APGII 2003; APGIII. 2009; Bayer and Kubitzki 2003) it is widely distributed as the largest member in the family, over tropical and subtropical regions especially in warm areas owing to its attractiveness as an ornamental plant (Mabberley 2017; Metcalfe and Chalk 1950). *Hibiscus* is a rapidly expanding paraphyletic genus with other genera nested within the Hibisceae tribe; which have a lack of unique shared derived characteristics (synapomorphies). The first revisions of the genus have been established as a broader circumscription by (Hochreutiner 1900), comprising about 197 species, subdivided into 12 sections. Nonetheless, the genus was divided into sections and segregated genera; the infrageneric classification of this genus needs further investigation. According to the recent revisions of this genus, the approximate number of species has been increased to 437 accepted species (Powo 2024). The genus has been recognized with an ethnobotanical significance due to its possessing of numerous bioactive metabolites (Fitrotunnisa et al. 2019; Hamrita et al. 2022; Liu et al. 2022; Vignesh and Nair 2018). Taxonomically, the *Hibiscus* genus is heterogeneous and exhibits a significant taxonomic complexity, adding more difficulties in distinguishing the traits among its species (Bayer et al. 1999; Blanchard Jr 2008; Fryxell 1980, 1997). Despite recent taxonomic studies based on molecular techniques being performed, many questionable marks in the genus delimitation were reported with no acceptable interpretations of the morphological variations (Pfeil et al. 2002; Pfeil and Crisp 2005). In general, morphology and anatomy were implemented to be the chief trends in plant systematics (Endress et al. 2000). Nevertheless, traditional taxonomic methods, reliant on morphological and anatomical characteristics, often encounter limitations when faced with cryptic species or instances of convergent evolution. These challenges have spurred a growing interest in alternative approaches, with chemotaxonomy in which the secondary metabolites are often specific to taxonomically related species, emerging as a promising avenue for providing a molecular perspective on species delineation. In this context, the main objective of this study is to discern the phenetic relationships between five *Hibiscus* species, namely: *Hibiscus* × *rosa-sinensis* L., *Hibiscus sabdariffa* L., *Hibiscus schizopetalus* (Mast.) Hook. f., *Hibiscus syriacus* L. and *Hibiscus tiliaceus* L. by the investigation of traditional morphological and anatomical characteristics as compared to the contemporary chemotaxonomy by: (i) describing leaf morphology (stomata types, trichomes diversity, epidermal cellular patterns, and leaf venation types); (ii) characterizing leaf and petiole anatomy, and (iii) analyzing phytochemical constituents of methanolic leaf extracts of the studied species. We aim to harness the morphological, anatomical, and phytochemical approaches to identify discrete character states that could be applied to the entire *Hibiscus* genus.

## Materials and methods

### Specimen collection and identification

Five species representing four sections of *Hibiscus* were collected from Egypt; collections localities as well as voucher deposition numbers are shown in Table 1. Identification and authentication of the specimens were carried out following standard literature **(**Bayer and Kubitzki 2003**)**. The voucher herbarium specimens were prepared and matched, for identification, against authentic ones at the Orman Botanical Garden, Giza, Egypt. The scientific names of the plants were verified according to the websites of the International Plant Names Index: www.ipni.org./ipni/query_ipni.html.

**Table 1:** The sections, vernacular names, and localities of the studied *Hibiscus* species

Voucher deposition No	Scientific name	Vernacular name	Sections according to Hochretiner (1900)	Locality
2633CH161,05–08-03–22	*H.* × *rosa-sinensis*	The rose of China	*Lilibiscus*	Orman Botanical Garden, Giza, Egypt
2639CH167,05–08-03–34	*H. sabdariffa*	Rosella	*Furcaria*	Kafr El-Shaikh Province, Egypt
2642CH170,05–08-03–37	*H. schizopetalus*	Fringed rose mallow	*Lilibiscus*	Orman Botanical Garden, Giza, Egypt
2647CH175,05–08-03–42	*H. syriacus*	Rose of Sharon	*Bombycella*	Orman Botanical Garden, Giza, Egypt
2665CH188,05–08-03–55	*H. tiliaceus*	Sea Hibiscus	*Azanza*	Orman Botanical Garden, Giza, Egypt

### Light microscopy (LM) investigations

The specimen collection was from July to September 2022. The collected mature leaves were fixated in F.A.A. [5 ml Formaldehyde (40%): 5 ml Glacial Acetic Acid: 90 ml Alcohol (70%)] and subsequently preserved in 70% ethyl alcohol until further analysis.

### Leaf architecture investigations

The fresh mature expanded leaves were decolorized by immersing the leaves in domestic bleach; sodium hypochlorite for 3 h, then being rinsed three times with tap water before being transferred to 50% ethyl alcohol and stained with 1% Safranine. Excess stain was washed with 50% alcohol, the leaves were dried and pressed between filter papers, and were examined and photographed with a dissecting microscope. The outcomes were described according to leaf architecture characters and terminologies (Ellis et al. 2009; Hickey 1973).

### Leaf epidermal characterization

The fresh and preserved developed mature leaves commonly from the 6th -10th nodes of the specimen branch were used; they were belled using nail polish on abaxial and adaxial surfaces and dried for approximately twenty minutes. The dried cast of the epidermal surface was separated with clear tape and mounted onto a glass slide. The epidermal cellular patterns, stomata types, and trichomes diversity were recorded and discussed on the adaxial and abaxial surfaces of the leaf.

### Leaf and petiole anatomy

Preserved leaves and petioles were prepared for paraffin wax embedding, sectioned by the microtome into 10–16 μm according to Johansen (1940). Serial transverse cross-sections of the petiole from proximal to distal zone were executed and stained with 2% Safranine. The stained sections were mounted in Canada balsam to a glass slide. The morphological and anatomical slides were observed and photographed at different magnifications using a bright field light microscope (LM) equipped with Scope Tek, scope photo 3.0 Digital Camera (CIT Nelian).

### Gas chromatography-mass spectrometry (GC–MS) analysis

Fresh healthy leaves for each species were separated and washed, shade-dried, and pulverized to powder. The powdered leaves (20 g) for each species were extracted with (125 ml) methanol of HPLC grade for 72 h under continuous stirring by Orbital Shaker at room temperature in tightly sealed conical flasks. Each extract was filtered using muslin cloth; the filtrates were collected and centrifuged. The supernatant was collected, the solvent was evaporated to 5 ml final volume, and then stored in tightly sealed dark vials at 4 °C for subsequent assay and analysis.

The analysis was carried out using a GC (Agilent Technologies 7890A) interfaced with a mass-selective detector (MSD, Agilent 7000) equipped with a polar Agilent HP-5 ms (5%-phenyl methyl poly siloxane) capillary column (30 m × 0.25 mm i. d. and 0.25 μm film thickness). The carrier gas was Helium with a linear velocity of 1 ml/min. The injector and detector temperatures were 200 ℃ and 250 ℃, respectively. The volume injected was 1 μl of the sample. The MS operating parameters were as follows: ionization potential 70 eV, interface temperature 250 ℃, and acquisition mass range 50–800. The identification of components was based on a comparison of their mass spectra and retention time with those of the authentic compounds and by computer matching with NIST and WILEY library as well as by comparison of the fragmentation pattern of the mass spectral data with those reported in the literature. The chemical composition of each extract was carried out at the Regional Center for Food & Feed (RCFF); Research Foundation in Giza, Egypt.

### Statistical analysis

The obtained data (42-character states of 21 attributes) from traditional approaches, furthermore (40) phytochemical bioactive constituents from GC–MS profiling technique of the leaf extract were separately constructed in a data matrix on Microsoft Excel and were subjected to Past4.09_32.exe software program (Tables S1, S2). The multistate characters were transformed into two-state characters in coding **(**Sneath and Sokal 1973**)**. Each of all characters was treated as a binary character in a data matrix; the presence coded 1, and the absence coded 0. Unweighted pair group method (UPGMA) phenogram clustering and Principal Component Analysis (PCA) were assayed to demonstrate the relationship and the variation among species, in addition to comparing the resulted dendrograms and assaying the weight of the characters.

## Results and discussion

### Leaf morphological characterization

**Fig. 1:**
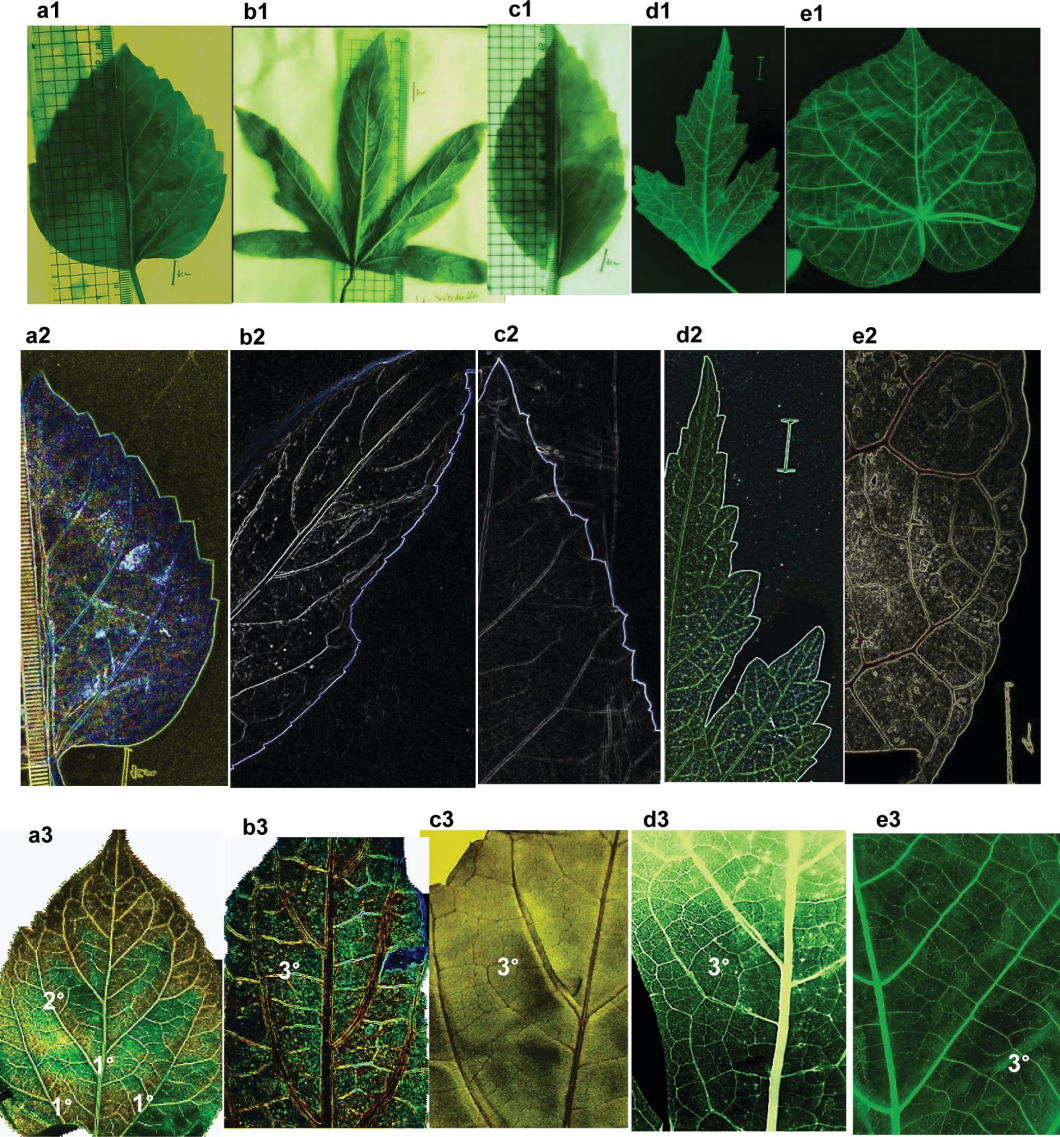
Leaf architectural characteristics of the studied *Hibiscus* species. *a1-3* *H.* × *rosa-sinensis*, *b1-3* *H. sabdariffa*, *c1-3* *H. schizopetalus*, *d1-3* *H. syriacus*, *e1-3* *H. tiliaceus*, *a1-e1* laminar shape, *a2-e2* marginal and apex configuration, *a3-e3* major venation pattern

**Fig. 2:**
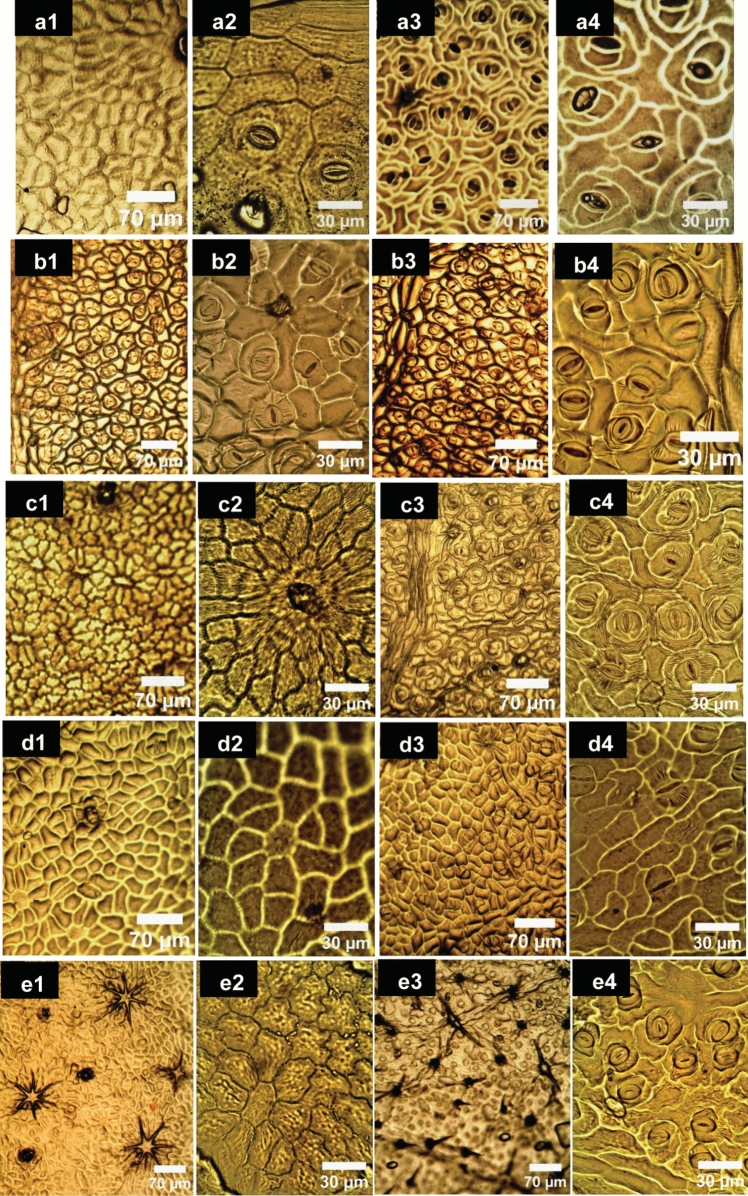
LM photographs of the leaf epidermal morphological characterizations of the five studied *Hibiscus* species. Revealing the cellular epidermal patterns and the stomata types on both leaf surfaces. *a1-4* *H.* × *rosa-sinensis*, *b1-4* *H. sabdariffa*, *c1-4* *H. schizopetalus*, *d1-4* *H. syriacus*, *e1-4* *H. tiliaceus*. a1, 2-e1, 2: adaxial surfaces, a3, 4-e3, 4: abaxial surfaces

**Fig. 3:**
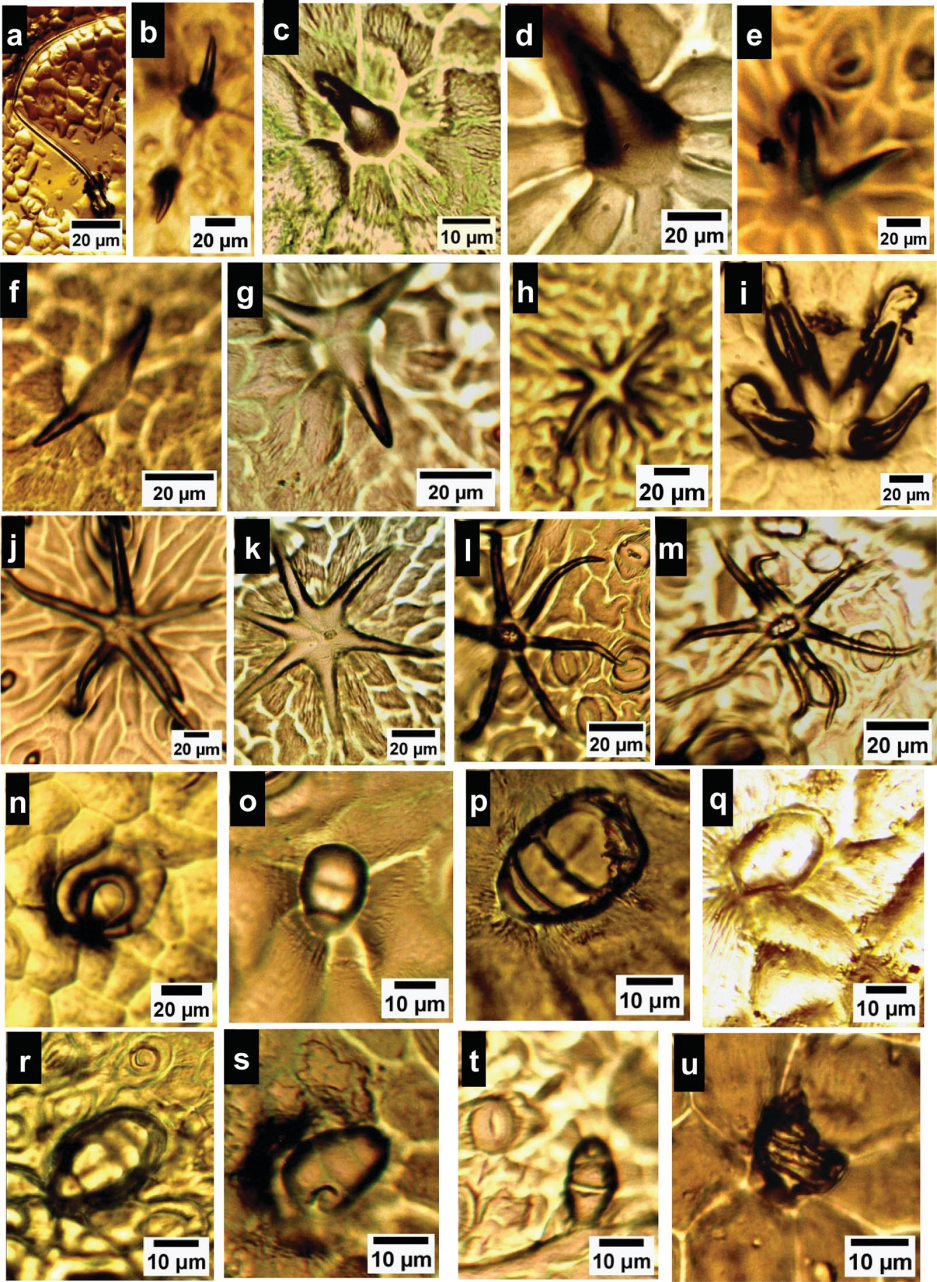
LM photographs of the trichome types recorded on the leaf of the studied *Hibiscus* species. **a**–**m** E-landular trichome types. **a** simple falcate type from *H. sabdariffa*, **b**–**d** conical type from *H. tiliaceus*, e, f: bi furcate type; erect standing and appressed types from adaxial of* H.* × *rosa-sinensis* and *H. tiliaceus*, respectively, g-m: 3–8 stellate hairs; **g**, **h**: 3 & 4 armed appressed types from adaxial surface of *H. tiliaceus*, **i**, **j**: 4-armed erect standing type and 6-armed appressed type from adaxial and abaxial surfaces of *H.* × *rosa-sinensis*, respectively. **k**–**m** 6 & 8 armed stellate hairs from *H. tiliaceus*; **k**: with the broad central portion on adaxial surface and **l**, **m** with the narrow central portion on abaxial surface. **n**-**u** Glandular trichome types at different developmental stages from single to multi-celled body. **n**–**t** sessile types and u: stalked type. **n**–**q** capitate-obovate glandular type; **n**–**p**, on *H.* × *rosa-sinensis* and **q** on *H. syriacus*. **r**–**t** ovate-elliptical glandular types from *H. tiliaceus*. **u** stalked broad elongated multicellular glandular type from *H. sabdariffa*

The micro-morphological features of the epidermis were investigated; the focus was on the most accurate detail traits including venation patterns, epidermal cellular patterns, anticlinal wall features, cuticular depositions, stomata types, and trichome diversity. In addition, qualitative macro-morphological characteristics of the leaf, including Laminar shape, margin type, apex shape, teeth tips, and leaf incisions were described as recognized character states for each of the studied species. The salient variations among the studied species were recorded (Tables 2, 3**, **Figs. 1, 2, 3**)**. Concerning the leaf architecture, venation patterns were reported to be genetically fixed and have been assayed as a precious taxonomic tool at different hierarchical levels, especially in the lack of reproductive parts such as flowers and fruits (Escalona and Buot 2023; Fuller and Hickey 2005; Masungsong et al. 2019). For the studied species, the major venation patterns of primary **(1°)**, secondary **(2°)**, and tertiary **(3°)** veins patterns were investigated. The salient comparative features among the studied species were recorded (Table 3, Fig. 1a–e). The basal actinodromous venation pattern of three primary veins; diverging radially from the leaf base is the only primary vein category in the five studied species. The diagnostic variances were reported in the secondary and tertiary vein categories, where festooned semi-craspedodromous of secondaries besides alternate percurrent tertiaries were the chief venation patterns in *H.* × *rosa-sinensis, H. sabdariffa, and H. syriacus* (Fig. 1a3-b3, d3). Festooned brochidodromous secondaries and mixed opposite alternate percurrent tertiaries are unique distinguishable traits for *H. tiliaceus* (Fig. 1e). Craspedodromous secondaries and alternate percurrent tertiaries are the veins categories in *H. schizopetalus* (Fig. 1c3). Equally, the epidermal characters were reported to be potential markers at different taxonomic levels; families, genera, and sometimes for infrageneric classifications of complex taxa (Celka et al. 2006; Hutchinson 1969; Metcalfe and Chalk 1950; Zhao et al. 2022)**.** Micromorphological investigations of the leaf epidermis revealed polygonal isodiametric-elongated regularly arranged cellular patterns on both surfaces. The cellular patterns exhibited a rosette-shaped arrangement; the central cell was surrounded by a ring of cells on the adaxial surface, while* H. sabdariffa* did not exhibit this arrangement due to the dense stomata frequencies (Fig. 2b1). The anticlinal walls are thick elevated and undulated, while periclinal walls are convex with striated cuticles. Notable variations between investigated species were recorded; separating the species from each other for example (a) Cuticular striations are prominent and continuous on both surfaces of* H. schizopetalus* (Fig. 2c**),** while in the remaining species is discontinuous, occasionally radiating from the base of trichomes and the guard cells to the subsidiary cells of the stomata complex. (b) The anticlinal walls undulation is straight on both surfaces of *H. sabdariffa* whereas, slightly sinuous in the remaining. Stomata are amphistomatic, with high condensation on abaxial surfaces, while adaxial stomata frequency reveals characteristic variance in its distributions. Stomata are densely distributed over the blade of* H. sabdariffa*, while in* H.* × *rosa-sinensis* and* H. schizopetalus* are arranged along the mid-rib veins while in *H. syriacus* and* H. tiliaceus* are moderately scattered over the blade. The most frequently occurring stomatal types on mature leaves were identified. These primarily included four types (anisocytic, anomocytic, paracytic, and brachyparacytic). Less frequent stomatal forms, such as hemiparacytic and staurocytic types, were noted but were considered as rare occurrences. To maintain clarity and consistency in species identification and delineation, these less frequent types were omitted from the analysis. The frequent types for each species were tabulated (Table 3).

**Table 2:** Recorded trichome types with their description and distribution

Trichome types			Description	Fig. 3	Occurrence
E-glandular type	Unicellular	Simple falcate	Long unicellular appressed hair is mainly represented on the vein courses	a	H2^*^
		Conical	Simple trichome with a pedestal as broad base and a tapering apex, protruding from specialized rose-shaped epidermal cells	b-d	H1, H3, H4, H5^*^
	Multicellular	Bifurcate	2-armed trichome, appressed or erect standing	e & f	H1, H3, H4, H5
		3–8 stellate	Multi-radiate of uniseriate rays with narrow or wide centers according to the number of rays. Rays appeared to be appressed or erect standing	g-m	H1, H3, H4, H5
Glandular types	Sessile	Capitate-obovate type	Multicellular at different division stages from single two-celled body to multi-celled one. The apical hemisphere is the largest	n-q	H1, H3, H4
		Ovate-elliptical type	Multicellular mainly of 3–4 celled body with small apical hemisphere cell	r-t	H2, H5
	Stalked	Elongated broad short stalked type	Multicellular body on short single celled stalk	U	H2

H1: *H.* × *rosa-sinensis*, H2: *H. sabdariffa*, H3: *H. schizopetalus*, H4: *H. syriacus*, H5: *H. tiliaceus*. H2^*^: the simple falcate hairs represent is the predominant e-glandular trichome type on both surfaces of *H. sabdariffa*. H5^*^: the conical type is predominant on abaxial in *H. tiliaceus*, besides stellate and bi-furcate types. On the other species the tress recorded e-glandular types are intermixed with each other's with high representation on the abaxial surface

**Table 3:** Morphological and anatomical characteristics of the leaf of the studied species

Characters	Species attributes	*H.* × *rosa-sinensis*	*H. sabdariffa*	*H. schizopetalus*	*H. syriacus*	*H. tiliaceus*
Laminar characteristics	Shape	Ovate	Elliptical	Elliptical	Elliptical	Cordate
	Margin type	Dentate-serrate	Serrate	Dentate-serrate	Dentate-serrate	Crenate
	Apex shape	Straight acute	Straight acute	Straight acute	Straight acute	Straight acute
	Teeth tips	Acute	Acuminate	Acuminate	Acute	Blunt
	Leaf incision	Unlobed	Palmiti-sect	Unlobed	Palmiti-fid	Unlobed
The major venation patterns	**1°**	Ba	Ba	Ba	Ba	Ba
	**2°**	Fsc	Fsc	C	Fsc	Fb
	**3°**	Ap	Ap	Ap	Ap	M/opp./alt
Cuticular deposition	Striation	Discontinuous	Discontinuous	Continuous	Discontinuous	Discontinuous
Anticlinal wall	Undulation	Slightly sinuous	Straight	Slightly sinuous	Slightly sinuous	Slightly sinuous
Stomata	Position	As	As	As	As	As
	Frequency on adaxial	Few	Dense	Few	Few	Few
	Distribution on adaxial	Arranged along mid ribveins	Over blade	Arranged along veins	Scattered over blade	Scattered over blade
	Frequency on abaxial	High	High	High	High	High
	Predominant	Ani	Ani/Para/Bp	Ani	Para	Para/Bp
E-glandular trichome	Position	Amphitrichomic	Amphitrichomic	Amphitrichomic	Amphitrichomic	Amphitrichomic
	Simple falcate	Intermixed	Intermixed	Intermixed	Intermixed	Not detected
	Conical	Intermixed	Not detected	Intermixed	Intermixed	Predominant
	Bi-furcate and stellate	Intermixed	Not detected	Intermixed	Intermixed	Intermixed
Glandular trichomes	Sessile	+	+	+	+	+
	Stalked	–	+	-	-	–
	Capitate-obovate	+	–	+	+	–
	Ovate- elliptical	–	+	–	–	+
	Elongated broad short stalked	–	+	–	–	–
Petiole cross-section	Outline in middle zone	Semi-terete	Semi-terete	Semi-terete	Semi-terete	Semi-terete
	Adaxial groove on proximal and distal	Shallow grooved	Grooved	Shallow grooved	Straight flattened	Straight flattened
	Pubescence	Pubescent	Pubescent	Pubescent	Pubescent	Pubescent
Petiole cortical layers	Epidermis	S/R	S/R	S/R	S/R	S/R
	Collenchyma	5–6	2–3	4–5	4–5	6–8
	Parenchyma	5–6	3–4	5–6	5–6	10–12
	Pith width in middle zone	Wide 12–13	Very wide 19–20	Wide 12–13	Narrow 6–8	Wide 10–12
Pericyclic fibers	Proximal	Separate patches	Separate patches	Separate patches	Separate patches	Separate patches
	Middle	Dissected ring	Dissected ring	Dissected ring	Dissected ring	Dissected ring
	Distal	Connected ring	Connected ring	Connected ring	Connected ring	Connected ring
Crystal idioblasts	Occurrence (druses)	Petiole & leaf	Petiole & leaf	Petiole & leaf	Petiole & leaf	Petiole & leaf
Epidermal secretory idioblasts	In petiole	Few	Few	Few	Few	Few
	In leaf laminar	Numerous	Numerous	Numerous	Numerous	Numerous
Secretory ducts	presence	-	+	-	+	+
Mucilage idioblasts	In petiole and leaf	+	+	+	+	+
	Density	Numerous	Numerous	Numerous	Few	Numerous
	Size	Medium	Medium	Medium	Medium	Large
Vasculature system	Main vbs	Three	Three	Three	Three	Three
	Type	Opened arc	Opened arc	Opened arc	Opened arc	Opened arc
	Accessory vbs	Three-four	Six-eight	Three-four	One	One
Midrib characteristics	outline	Biconvex	Biconvex	Biconvex	Biconvex	Flat-convex
	Uc	7–8	7–8	5–6	6–7	4–5
	Lc	5–6	4–5	4–5	5–6	4–5
	Up	10–11	10–11	12–14	4–5	10–11
	Lp	3–4	3–4	3–4	3–4	4–5
	Sf	+	+	+	+	+

*Ap* Alternate percurrent, *Accessory vbs* Accessory vascular bundles, *As* Amphistomatic. *Ba* Basal actinodromous, *Ani* Anisocytic, *Bp* brachyparacytic, *C* Craspedodromous, *Fb* Festooned brochidodromous, *Fsc* Festooned semi-craspedodromous, *M/opp/alt* Mixed opposite alternate percurrent, *Para* Paracytic, *Sf* Sclerenchyma fibers in leaf, *S/R*  Singular/Radially elongated epidermal cells, *Uc* upper collenchyma, *Lc* lower collenchyma, *Up* upper parenchyma, *Lp* lower parenchyma, ( +): detected, (-): not detected

Rao and Ramayya (2008) studied stomatogenesis and its organographic distribution in ten species of the genus *Hibiscus* and documented the anisocytic type as the most frequent type. In the present study, anisocytic stomata is the predominant type in *H.* × *rosa-sinensis* and* H. shizopetalous*, the paracytic type is predominant in the other three species, together with brachyparacytic, in* H. tiliaceus* and anisocytic and brachyparacytic in *H. sabdariffa*. In terms of trichome diversity, Bayer and Kubitzki (2003) discriminated the order Malvales based on the e-glandular trichomes. Shamsuddin et al. (2022) studied trichomes diversity in Malvaceae subfamilies, Bombacoideae and Helicteroideae and authenticated its taxonomic value in species identification and delineation. Raja Rao (1991) studied the structure and organographic distribution of trichomes on different organs in ten species of *Hibiscus*. Shaheen et al. (2009) studied the foliar trichome diversity in seven species of the genus* Hibiscus*, the discussed types were not carefully examined, and their interpretation was ambiguous. For the investigated species, both glandular and e-glandular types are intermixed on the leaf surfaces, however, there are variations in its morphology and density that can be harnessed in species recognition. The recorded types are collectively illustrated, and the predominant characteristic types of each species are tabulated (Table 2**, **Fig. 3). E-glandular types were subdivided into two main types a) unicellular type: which encompasses simple long falcate hairs that represent the predominant and main type of e-glandular trichomes in *H. sabdariffa* (Fig. 3a), in addition to short conical trichomes that are the chief frequent type in *H. tiliaceus* (Fig. 3b). (b) Multicellular types are categorized into two sub-categories; 2-armed (bifurcate) type and 3–8 stellate type. On the other hand, glandular trichomes were investigated at different developmental stages diverse from single-celled secretory structures to multicellular ones. It is categorized morphologically into (a) Capitate-obovate sessile glandular type, the predominant in *H.* × *rosa-sinensis*, *H. schizopetalus* and *H. syriacus*. (b) Ovate-elliptical sessile glandular type was recorded in* H. tiliaceus* and* H. sabdariffa* condensed along the main veins (Fig. 3r-t). (c) Stalked elongated broad multicellular glands, observed only in *H. sabdariffa* (Fig. 3u). Last, but not least, the foliar epidermal surface was meticulously surveyed morphologically to obtain constant and precise traits that can be relied upon for more accurate species delineation and for enhancing our understanding of plant diversity and evolution.

### Leaf and petiole anatomical characterization

**Fig. 4:**
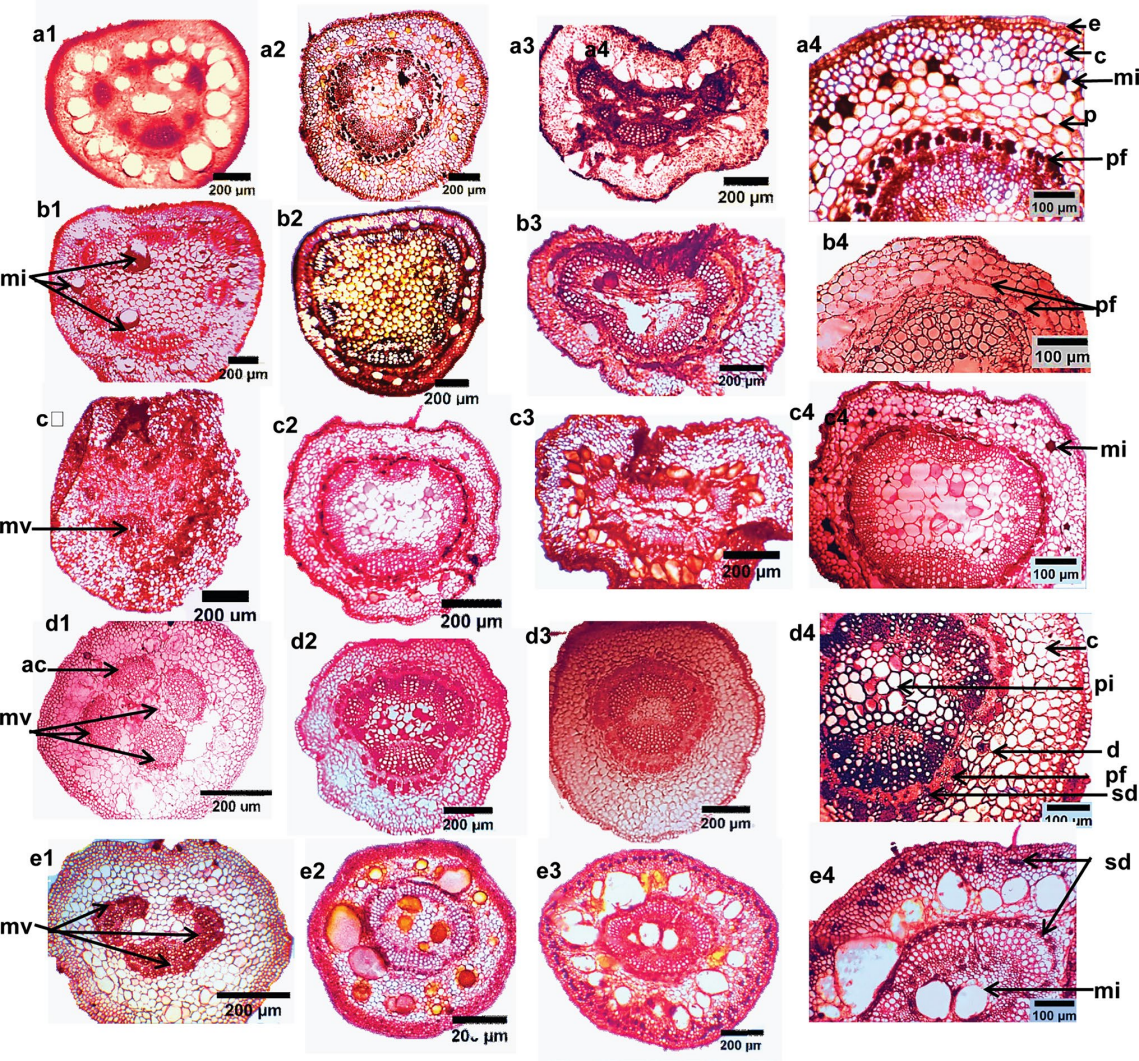
LM of transverse serial sections of the petioles of ***Hibiscus*** species. *a1-e1* proximal zone, *a2-e2* middle zone, *a3-e3* distal zone, *a4-e4* finer structural details of petiole middle zone. *a1-4* *H.* × *rosa-sinensis*, *b1-4* *H. sabdariffa*, *c1-4* *H. schizopetalus*, *d1-*4 *H. syriacus*, *e1-4* *H. tiliaceus*. *ac* accessory vascular bundle, *ag*: adaxial groove, *c*: collenchyma, *d*: druses, e: epidermis, mi: mucilaginous idioplasts, mv: main vascular bundles p: parenchyma, pf: pericyclic fibers, pi: pith, sd: secretory ducts, si: secretory idioblasts

**Fig. 5:**
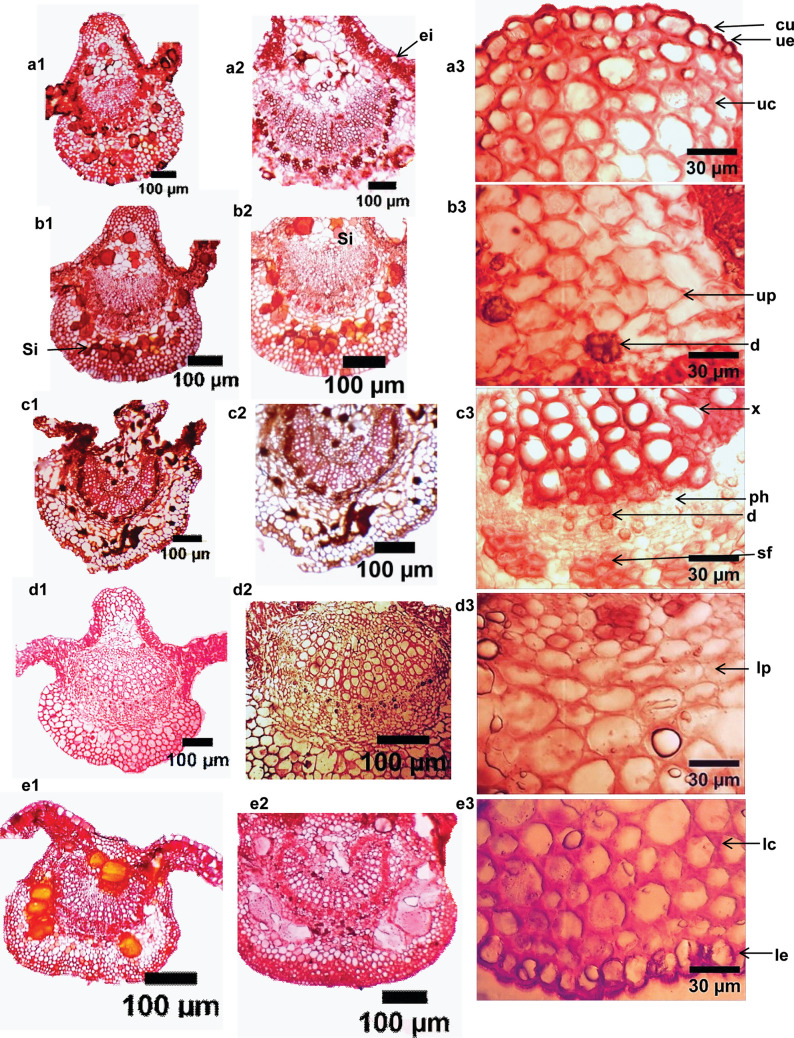
L.M. photographs of the leaf anatomical sections of *Hibiscus* species. *a1, 2* *H.* × *rosa-sinensis*, *b1, 2* *H. sabdariffa*, *c1, 2* *H. schizopetalus*, *d1, 2* *H. syriacus*, *e1, 2* *H. tiliaceus*, *a3-e3* The magnified structural characteristics of mid-rib cross sections of *H. syriacus* at 400x. *cu* cuticle, *d* druses, *ei* epidermal secretory idioblasts, *le* lower epidermis, *lc* lower collenchyma, *lp* lower parenchyma, *ph* phloem, *sd* secretory ducts, *sf* sclerenchyma fibers, *si* secretory idioblasts, *ue* upper epidermis, *uc* upper collenchyma, *up* upper parenchyma, *x* xylem

Anatomy was assayed to be the backbone of plant systematics and has long been widely used in the recognition and evaluation of taxonomic status as well as relationships of taxa of flowering plants, effective in phylogeny and evolution (Beck 2010; Cutler et al. 2007; Endress et al. 2000; Solereder 1908). Singh (1981) and Singh (2019) documented that the vegetative and floral anatomy is significant in solving taxonomic problems at different hierarchical levels. In this regard, the petiolar and leaf anatomy were assayed to have significance at inter-generic and familial circumscription (Akinsulire et al. 2018; Olowokudejo 1987), particularly the arrangement and structure of vascular bundles in the species and sections delineation (Noor et al. 2023). In this context, the serial sectioning of the petiole of the five studied species along their lengths; proximal, middle, and distal were investigated and photographed (Fig. 3). The petioles are featured by abundant e-glandular trichomes and few glandular ones, condensed on their adaxis. The outline is semi-terete along its length in *H. syriacus* and *H. tiliaceus*. The variances were recorded on the adaxial portion in proximal and distal zones, in *H.* × *rosa-sinensis* and* H. shizopetalous*, it is shallowly grooved, while in *H. sabdariffa* is sunken grooved. The middle zone of the petiole was reported to be the most stable zone for comparative purposes, the structural characteristics along this zone are the same with trace variations that can be harnessed for species delineation. Consequently, this zone was considered the target zone for the comparison between the studied species. The epidermis consists of uniseriate thick cutinized radially elongated epidermal cells. Cortical tissues were numerically described; the number of tissues for each species was tabulated (Table 3) and previewed (Fig. 4). Pith width exhibits variance between the species, in* H. sabdariffa* is very wide; 19–20 cell layers thickness, wide in* H.* × *rosa-sinensis, H. shizopetalous* and* H. tiliaceus*; 10–13 cell thickness, while is narrow in* H. syriacus*; 6–3 cell thickness. The central stele is ectophloic eustele of collateral vascular bundles, enclosed by groups of separated pericyclic fiber patches at the proximal and dissected ring at the middle, which increased gradually and became a connected ring over the ring of the vascular bundles toward the distal zone. One of the most salient recorded features in the serial anatomical section of the petiole is the vasculature traces arrangement. This feature has been reported to provide diagnostic systematic features at any taxonomic rank (Metcalfe and Chalk 1979). The number of main vasculature traces is three opened arc vasculature arrangement of separate vascular bundles in the proximal zone; the main large abaxial and two laterals, the number increased upwardly even in the middle zone by additional accessory vascular bundles in between the main ones. The numbers of accessory vascular bundles are varied; three-four in* H.* × *rosa-sinensis* and* H. shizopetalous*, one In* H. syriacus* and* H. tiliaceus*, while in* H. sabdariffa* there are six-eight additional ones. The crystal idioblasts were represented as calcium oxalate crystals (druses); abundant in proximal cortical tissue, and within the leaf mesophyll and the mid-rib region in all species. Internal secretory structures were documented to have systematic significance at tribe rank and in identifying boundaries for taxa circumscription (Raghu 2015). The internal secretory structures of the studied species were distributed in constant positions in petiolar and laminar anatomical cross-sectioning. However, there are various types with various densities between the studied species. Three types are detected. (a) Epidermal secretory idioblasts; small rounded, radially arranged cells in alternate patterns within the epidermal cells, few in petiole and numerous in epidermal layers of mesophyll. It is represented in the five studied species (Fig. 4a2). (b) Mucilaginous idioblasts are abundant and well represented in petiole parenchyma and pith regions, in addition, parenchyma of mid-rib and mesophyll of lamina as large-sized cells, however, in *H. syriacus* it is differentiated by the fewest density when compared with the other species (Figs. 4, 5d). Furthermore,* H. tiliaceus* is characterized by the largest-sized mucilaginous cells (Figs. 4, 5e). The mucilaginous cells In* H.* × *rosa-sinensis*,* H. schizopetalus,* and* H. sabdariffa* are medium-sized. **c**) Secretory ducts/canals were investigated connected to the vascular system within the phloem of vascular bundles in* H. sabdariffa*,* H. syriacus* and* H. tiliaceus*. Furthermore, anastomoses of secretory canals are detected in the petiole collenchyma of *H. tiliaceus* (Fig. 4e4). One of the most interesting structural facts noticed through the serial sectioning of the petioles is that the transition zone of the leaf at the point of attachment with the petiole has 3 main vascular bundles that represent the 3 main vasculature traces at the proximal of the petiole and that confirming the tri-costate venation pattern of the leaf of the studied species.

On the other hand, the leaf anatomy investigations demonstrated that the leaf laminar is dorsiventral; mesophyll of single-layered palisade and 3–4 spongy layers. The midrib characterizations of the studied species revealed precious taxonomic traits that could be exploited for the separation of closely related species. The salient anatomical attributes were tabulated (Table 3, Fig. 5). The midrib is prominently conspicuous on the abaxial surfaces of the studied species, while on adaxial exhibits some variations. In *H. tiliaceus,* the midrib is flat on the adaxial, so its outline is flat-convex. The remaining species have a bi-convex outline. The cortical tissues, upper and lower of the midrib region were numerically described; the number of each tissue per species was tabulated (Table 3) to add quantitative attributes for further species identification. Sclerenchyma fiber batches were detected around vascular bundles. Herein, the structural characteristics of the leaf in addition to the serial sections of the petiole added a clear understanding of the internal structure of those organs and consequently introduced a vivid interpretation of the obtained data thus introducing strict and constant traits that can be utilized for the studied species identification and delineation.

### Phytochemicals profiling of the methanolic extracts of the studied species

**Fig. 6:**
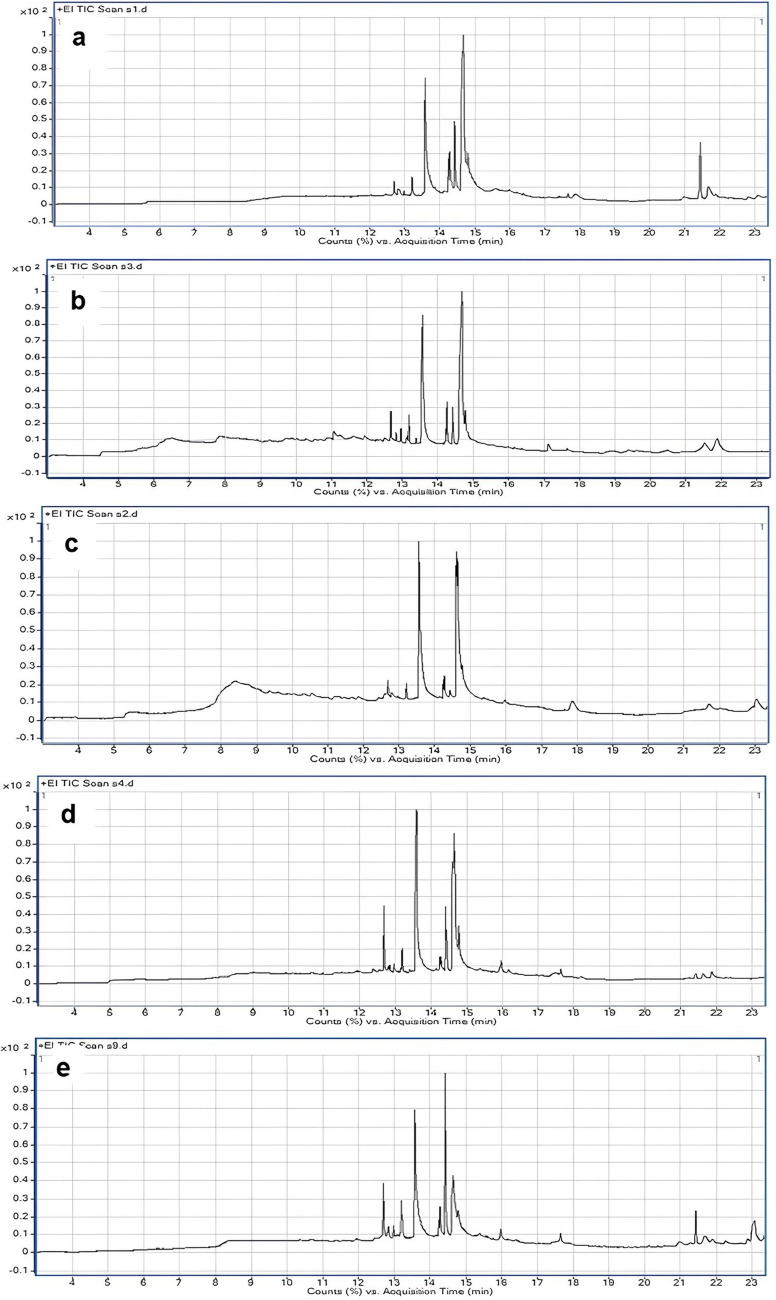
Gas chromatograms of methanol leaf extracts of the five studied *Hibiscus* species** a: ***H.* × *rosa-sinensis*, **b*:**** H. sabdariffa,*** c*:**** H. schizopetalus*, **d*:**** H. syriacus,* **e*:**** H. tiliaceus*

**Fig. 7:**
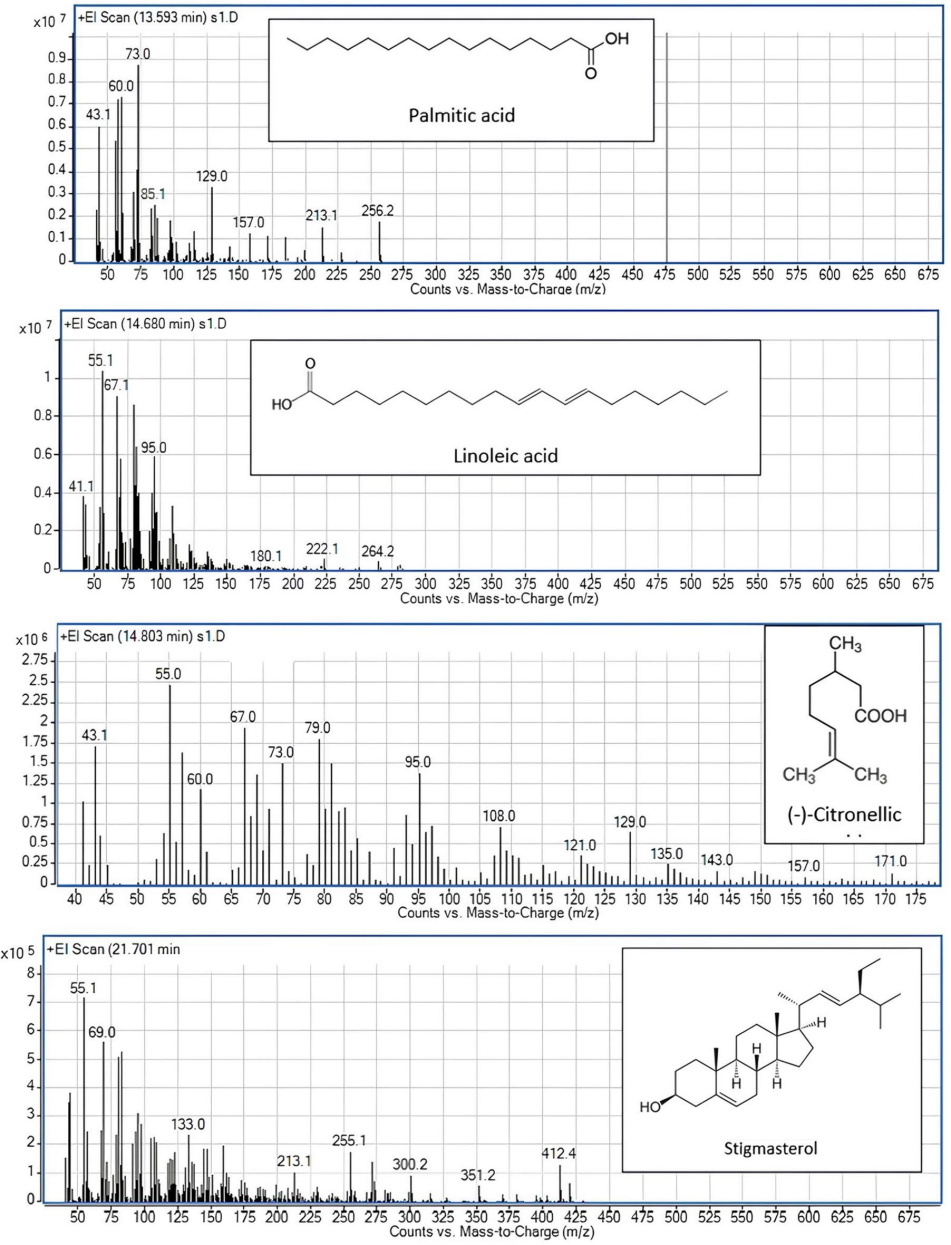
The mass spectra of some common compounds in the five studied species

Application of Gas Chromatography-Mass Spectrometry (GC–MS) as a combined analytical technique that supports the traditional approaches in plant taxonomy significantly enhances the classification and identification of plant species based on their secondary metabolite’s similarity and dissimilarity (Olivia et al. 2021; Singh 2016). This powerful analytical technique combines the separation capabilities of gas chromatography with the detection and identification abilities of mass spectrometry thus enabling the detection of unique chemical markers or compounds present in different plants, thereby assisting in distinguishing among the different species, subspecies, or varieties that might otherwise appear morphologically similar. Furthermore, GC–MS allows for the comparison of chemical profiles, facilitating the understanding of evolutionary relationships, exploring new trends of plant taxonomical studies, contributing significantly to the field of plant taxonomy. In this regard, the phytochemical screening of the methanol-based extract of the leaves of the five studied species of *Hibiscus* was analyzed. Specifically, the members of family Malvaceae were documented by their mucilaginous nature and their ability to produce numerous bioactive secondary metabolites with potential medicinal properties (Adusei 2020; Vignesh and Nair 2018). For the studied species, the traditional applied approaches, anatomy and morphology confirmed the excretory ability of these plants attributed to the diversity in internal and external excretory compositions. Raghu et al. (2019) applied preliminary histochemical tests on different organs of selected species of the genus *Hibiscus* to reveal their metabolic chemical classes**. **Olivia et al. (2021) analyzed the phytochemical profiling of selected species of *Hibiscus* for medicinal applications. The majority of analyzed chemical compounds reveal pharmacological properties of antioxidant, cytotoxic, antimicrobial, anti-diabetic, anti-tumor, anti-inflammatory and anti-obesity activities. The phytochemical screening of the leaf methanolic fraction of the five studied species of *Hibiscus* identified 40 chemical constituents with profitable biological activities. Figures 6 and 7, display the chromatograms and the mass spectra of the most predominant compounds. The recorded compounds were categorized as fatty acids, terpenoids, flavonoids, steroids, alkaloids, phenols, and glycosides. Fatty acids and terpenoids are the chief products followed by flavonoids then steroids. Alkaloids, phenols, and glycosides register the lowest distribution (Table 4). Ten fatty acids were analyzed; nine of them were detected in* H. sabdariff*a; five of them (No. 3, 6, 7, 12 and 31) are unique for this species and the remaining four (No. 13, 14, 15 and 20) are common in the five studied species. Palmitic and linoleic fatty acids were represented in high percentages in the studied species. Alkaloid and phenolic compounds are marker compounds for *H. sabdariffa*; they were analyzed as xanthine (2.6%) and trans-tris-methoxy-resveratrol (0.43%), respectively. In addition, cannabidiolic acid is detected in* H. syriacus*,* H. tiliaceus* and* H.* × *rosa-sinensis* in area percentage (2.62%), (0.47%) and (0.45%), respectively. Thus, it could be used as a key marker for* H. syriacus*. Alkaloid and phenolic compounds are known for their role in anti-herbivore defense strategies and may function to regulate microbial activity on the leaf surface (Pratyusha 2022). The leaf, stem, and calyces of *H. sabdariffa* were reported to be a potential source of antioxidants and many bioactive compounds (Adusei 2020). D-Glycero-d-galacto-heptose monosaccharide was detected in* H. schizopetalus* and* H. sabdariffa* with area percentages (34.03%) and (6.66%), respectively. It is worth mentioning that there is a high similarity in the phytochemical profiling of the studied species. For example, sixteen common compounds were reported, nine of them were predominant and represented in high area percentages in the studied species; those compounds are (s)-(-)-citronellic acid, palmitic acid, linoleic acid, (-)-citronellol, phytol, (-)-catechin gallate, methyl palmitate, isolongifolol and 18α-glycyrrhetinic. Thus, these metabolites could be used as chemotaxonomic markers at the genus level. Eleven distinguishable compounds are restricted only to* H. sabdariffa*, this species is separated chemically from the others. On the other hand, the lack of certain phytochemicals; that are otherwise detected in the other taxa at the same retention times may be considered as chemotaxonomic guides for some species. For example, the absence of apigenin 8-C-glucoside, retinoic acid (vitamin A) and spinacene may be used as characteristic features for* H. schizopetalus* and the absence of β-Sitosterol and stigmasterol could be used as a diagnostic character for* H. sabdariff*a. Apigenin 8-C-glucoside is the only analyzed glucoside, registering the highest area percentage in* H. syriacus* (3.14%), then *H. sabdariffa* (1.66%),* H.* × *rosa-sinensis* (1.09%) and finally *H. tiliaceus* (0.69%). α-Tocopherol (vitamin E), one of the most important naturally occurring antioxidants, assessing in inhibiting tumors and preventing diabetes complications (Lee and Han 2018), is characteristically represented in *H.* × *rosa-sinensis* (2.37%) and *H. schizopetalus* (0.41%).

**Table 4:** GC–MS phytochemicals profiling of methanol extracts for the five studied *Hibiscus* species

No	Compound Name	Molecular formula	Chemical class	RT	Area %				
					**H1**	**H2**	**H3**	**H4**	**H5**
1	d-Glycero-d-galacto-heptose	C_7_H_14_O_7_	Monosaccharide	8.3	**-**	6.66	34.03	**–**	**–**
2	L-Glucose	C_6_H_12_O_6_	Monosaccharide	9.3	**-**	1.8	**–**	**–**	**–**
3	14-Pentadecenoic acid	C_15_H_28_O_2_	Fatty acid	10.9	**-**	1.45	**–**	**–**	**–**
4	Xanthinin	C_17_H_22_O_5_	Alkaloids	11.09	**-**	2.6	**–**	**–**	**–**
5	α-Methylionol	C_14_H_22_O	Terpenoids	11.2	**-**	1.83	**–**	**–**	**–**
6	β-Hydroxydodecanoic acid	C_10_H_20_O_3_	Fatty acid	11.64	**-**	1.5	**–**	**–**	**–**
7	α,β-Gluco-octonic acid lactone	C_8_H_14_O_8_	Fatty acid	11.97	**-**	1.42	**–**	**–**	**–**
8	3',4',7-Trimethylquercetin	C_18_H_16_O_7_	Flavonoid	12.48	0.6	0.63	0.65	0.59	0.62
9	Phytol	C_20_H_40_O	Terpenoids	12.7	1.05	1.94	0.79	4.4	5.71
10	3-Methylkaempferol	C_16_H_12_O_6_	Flavonoid	12.8	0.86	0.48	0.67	0.84	1.11
11	10-Octadecenal	C_18_H_34_O	Hydrocarbon	12.98	0.44	0.75	0.37	0.66	0.74
12	cis-10-Nonadecenoic acid	C_19_H_36_O_2_	Fatty acid	13.13	**-**	0.67	**–**	**–**	**–**
13	Methyl palmitate	C_17_H_34_O_2_	Fatty acid	13.2	1.65	1.4	0.94	1.46	3.16
14	Oleic Acid	C_18_H_34_O_2_	Fatty acid	13.51	0.45	0.41	0.62	0.64	0.38
15	Palmitic acid	C_16_H_32_O_2_	Fatty acid	13.6	17.69	18.78	19.54	28.61	28.08
16	Flavonol 3',4',5,7-OH,3-O-araglucoside	C_27_H_30_O_15_	*Flavonoid*	13.87	0.49	0.64	**–**	**–**	0.88
17	Squalene	C_30_H_50_	*Hydrocarbon*	14.11	0.52	0.49	0.66	2.11	1.08
18	Isolongifolol	C_15_H_26_O	Sesquiterpenoid	14.25	2.18	0.52	1.02	1.38	2.21
19	(-)-Citronellol	C_10_H_20_O	Terpenoids	14.42	2.35	2.48	1.46	1.38	17.19
20	Linoleic acid	C_18_H_32_O_2_	Fatty acid	14.67	5.13	2.73	0.48	5.32	12.21
21	(S)-(-)-Citronellic acid	C_10_H_18_O_2_	Terpenoids	14.8	39.75	32.94	24.35	31.81	2.21
22	Retinoic acid	C_20_H_28_O_2_	Fatty acid	15.6	1.74	**-**	4.95	4.78	0.81
23	18α-Glycyrrhetinic acid	C_30_H_46_O_4_	Terpenoids	15.97	3.12	1.71	0.43	2.77	1.95
24	DELTA.9-Tetrahydrocannabinol	C_21_H_30_O_2_	Terpenoids	16.24	1.47	0.46	-	2.31	0.57
25	Cannabidiolic acid	C_22_H_30_O_4_	Terpenophenolic compound	16.38	0.45	-	-	2.62	0.47
26	trans-Trismethoxyresveratrol	C_17_H_18_O_3_	Polyphenol	17.13	**-**	0.43	**-**	**-**	**-**
27	Apigenin 8-C-glucoside	C_21_H_20_O_10_	Flavonoid 8-c-glycosides	17.4	1.09	1.66	-	3.14	0.69
28	Pregnan-20-one	C_21_H_34_O	Steroid	17.65	0.5	0.7	0.49	0.73	0.91
29	α-Tocopherol	C_29_H_50_O_2_	Vitamin E	17.9	2.37	-	0.41	**–**	**–**
30	β-Guaiene	C_15_H_24_	Sesquiterpenes	18.9	**–**	1.56	**–**	**–**	**–**
31	Arachidonic acid methyl ester	C_21_H_34_O_2_	Fatty acid	19.37	**–**	0.83	**–**	**–**	**–**
32	β-Sitosterol	C_29_H_50_O	Steroid	20.11	0.87	-	3.43	0.99	0.39
33	Isopatchoulenone	C_15_H_22_O	Terpenoids	20.47	**–**	1.19	**–**	**–**	**–**
34	Campesterol	C_28_H_48_O	Steroid	21.06	1.34	0.54	**–**	**–**	1.59
35	Spinacene	C_30_H_50_	Terpenoids	21.45	7.4	2.77	**–**	0.51	4.11
36	Stigmasterol	C_29_H_48_O	Steroid	21.7	3.98	-	0.4	0.65	2.38
37	(-)-Catechin gallate	C_22_H_18_O_10_	Flavans	21.8	0.99	4.91	1.75	1.44	0.96
38	5,7,4'-Trimethoxyisoflavone	C_18_H_16_O_5_	Flavonoid	22.28	0.54	0.49	0.95	0.87	1.12
39	Cyanin cation	C_27_H_31_O_16_^+^	Anthocyanin cation	22.8	0.97	-	1.63	**–**	1.19
40	Isoorientin	C_21_H_20_O_11_	Flavone	23.13	**–**	0.65	**–**	**–**	7.28

*H1* *H.* × *rosa-sinensis* L*.*, *H2* *H. sabdariffa*, *H3* *H. schizopetalus*, *H4* *H. syriacus*, *H5* *H. tiliaceus*, *RT* Retention time

### Cladistic analysis.

**Fig. 8:**
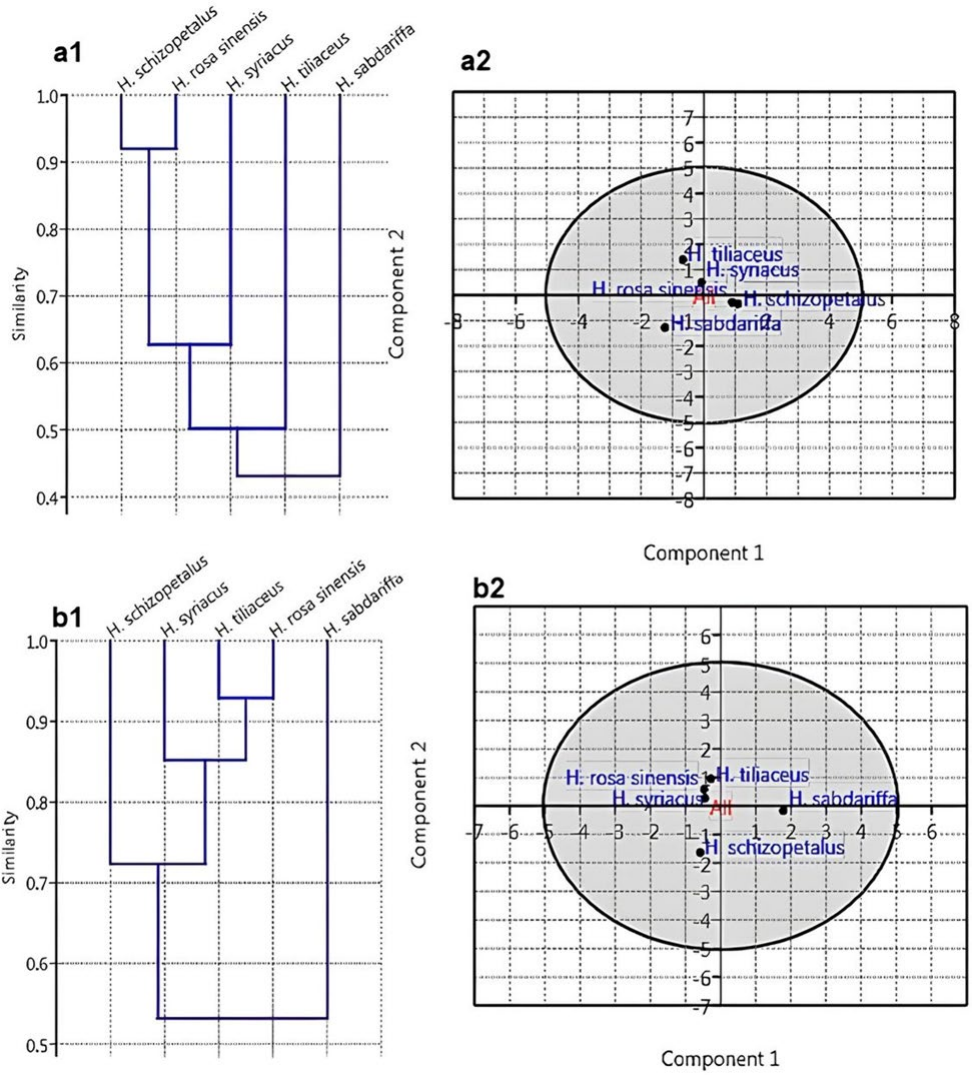
Numerical clustering of the five *Hibiscus* species. **a1**, **a2** Similarity clustering of the species based on the morphological and anatomical attributes. **b1**, **b2** clustering of the studied species based on phytochemicals profiling for their methanolic leaf extracts using GC–MS. **a1**, **b1** UPGMA analysis & **a2**, **b2** PCA plotting analyses

Multivariate clustering based on the UBGMA method using the Jaccard coefficient (Sneath and Sokal 1973) and Principal component analysis (PCA) were employed. PC1 for the X-axis, representing the most variable component, and PC2 for the Y-axis, representing the second component in the variability for 42 comparative character states of 21 traits based on traditional morphology and anatomy approaches (Table S1), as well as for 40 comparative metabolic bioactive components resulting from GC–MS profiling of the examined species (Table S2). These methods were constructed to discern the most variable discrete character states that were harnessed in building phenetic relationships through the similarity indices from the set of sharing characters of the studied species. The worthy characteristics constructed in the data matrix based on the traditional morphology and anatomy investigations are leaf venation patterns, epidermal micromorphological characteristics, stomata, trichomes petiole outline, adaxial groove features, vasculature traces arrangement and midrib characteristics. Both, morpho-anatomical-based dendrogram (Fig. 8a1) and chemotaxonomy-based dendrogram (Fig. 8b1), separate the five studied species into two clusters with* H. sabdariffa* separated as outlier species and represent a first cluster and the remaining four species (*H.* × *rosa-sinensis, H. shizopetalous, H. syriacus* and* H. tiliaceus.)* are separated as a second cluster at variant distances in its similarity indices. Specifically, the traditional morphology and anatomy based dendrogram (Fig. 8a1) revealed that the studied species had an average taxonomic distance of 0.425. At this point, there were two clusters, the first cluster encompassed* H. sabdariffa* and the second cluster was differentiated into* H. tiliaceus* in one group and* H. shizopetalous, H.* × *rosa-sinensis, H. syriacus* in another group at 0.500, which was then separated into two sub-clusters at 0.625 with the* H. shizopetalous* and* H.* × *rosa-sinensis* represented in one group with a similarity index of about 0.920. The PCA plotting analysis in (Fig. 8a2) authenticates the same result by constructing taxonomic relationships between the five studied species which can be summarized as follows:* H.* × *rosa-sinensis* and* H. shizopetalous* share a common set of characters and are closely related to* H. syriacus* then to* H. tiliaceus* and finally to* H. sabdariffa*. On the other hand, the chemotaxonomy-based dendrogram (Fig. 8b1) indicated that the average taxonomic distance of the studied species is 0.525. At this level, there were two clusters, the first cluster represented *H. sabdariffa* and the second cluster was differentiated into* H. shizopetalous* in one group and* H. syriacus, H. tiliaceus* and* H.* × *rosa-sinensis* in another group at 0.725, which was then separated into two sub-clusters at 0.850 with the* H. tiliaceus* and* H.* × *rosa-sinensis* represented in one group with a similarity index of about 0.950. Similarly, the PCA plotting analysis (Fig. 8b2) demonstrated that *H.* × *rosa-sinensis* and *H. tiliaceus* share a common set of characters and are closely related to* H. syriacus* then to *H. shizopetalous* and finally to* H. sabdariffa***.** It is noteworthy that *H. schizopetalus* is the farthest from * H. sabdariffa* in both traditional morphology and anatomy and chemotaxonomy approaches. The loading scores of each variable with high loading scores (either positive or negative) on the first principal component (PC1) for the morphoanatomical attributes and the phytochemical variables (Figs. S1, S2) indicate how strongly correlated they are. Most of the selected attributes exhibit variation between the studied species and reflect a reliable relationship that can be harnessed in species identification and delimitation at different hierarchical levels.

## Conclusion

The present study disclosed comparative investigations for the implementation of traditional morphological and anatomical approaches compared to the contemporary chemotaxonomy on the leaves of five *Hibiscus* species, namely:* H.* × *rosa-sinensis*, *H. sabdariffa*,* H. schizopetalus*,* H. syriacus* and* H. tiliaceus* to assess the degree of variations in the set of shared characters. The similarity coefficient among the studied species established a phenetic circumscription that aligns with previous phylogenetic studies, thus validating the taxonomic significance of the reported characters. The morphological and anatomical characteristics of the studied species included leaf venation patterns, epidermal micromorphology, stomata, trichomes diversity, petiole outline, adaxial groove features, vasculature traces arrangement, and midrib characteristics. On the other hand, in the chemotaxonomy approach, the leaf-based methanolic extracts of the studied species were analyzed by Gas Chromatography-Mass Spectrometer (GC–MS) to estimate their secondary metabolites’ similarity. The results of both chemotaxonomy and traditional taxonomy exhibited a remarkable agreement in the delineation of the five studied species with the* H. sabdariffa* separated in one cluster and the remaining four species in another cluster with variant distances in its similarity indices. It can be concluded that, the integration of the structural features with phytochemicals profiling data identified discrete distinguishing features that sharply differentiated between the studied species and could be applied to a wide range of species and genera for accurate identification and classification.

## Supplementary Information

Below is the link to the electronic supplementary material.

Supplementary file1 (PDF 436 KB)

## Data Availability

The data supporting this study's findings are available from the corresponding author, upon reasonable request.
