# Supplementary material for: Chemotaxonomy compared to morphological and anatomical taxonomy of five Hibiscus species
Source: J Plant Res. 2024 Aug 15;137(6):967–84. doi: 10.1007/s10265-024-01566-9 (PMC11525320; doi:10.1007/s10265-024-01566-9)
Supplement: Supplementary file 1 — Supplementary file1 (PDF 436 KB) [file 10265_2024_1566_MOESM1_ESM.pdf]

Supporting Information

Chemotaxonomy compared to morphological and anatomical taxonomy of five *Hibiscus* species

Hala M. E. Abdelfattah,*[1] Hussein A. Hussein,[1] Samir S. Teleb,[1] Marwa M. El-Demerdash,[1] Nelly M. George[1]

[1]Department of Botany and Microbiology, Faculty of Science, Zagazig University, Zagazig 44511, Egypt

*Corresponding author's email address: halaammar91@gmail.com

Contents

Description		Page No.
Table S1	Data matrix of morphological and anatomical characters and their states used in numerical analysis	S2
Table S2	Data matrix of GC-MS phytochemical compounds applied in numerical analysis	S3
Fig. S1	The loading scores of each variable (41 character states, Table S1) from traditional morphoanatomical characters on the most variable principal component (PC1).	S4
Fig. S2	The loading scores of each variable (40 phytochemical compounds, shown in Table 4) from GC-MS analysis of the methanolic leaf extract using the most variable principal component (PC1).	S4

Table S1 Data matrix of morphological and anatomical characters and their states used in numerical analysis

No	Characters	Character states	*H. rosa sinensis*	*H. sabdariffa*	*H. schizopetalus*	*H. syriacus*	*H. tiliaceus*
1	Venation patterns	Ba.1°	1	1	1	1	1
		C 2°	0	0	1	0	0
		Fsc 2°	1	1	1	1	0
		Fb 2°	0	0	0	0	1
		Ap 3°	1	1	1	1	0
		M/opp./alt.3°	0	0	0	0	1
2	Cuticular striations (Cs)	Continuous	0	0	1	0	0
		Discontinuous	1	1	0	1	1
3	Anticlinal wall (Aw)	Straight	0	1	0	0	0
		Slightly sinuous	1	0	1	1	1
4	Stomata	Amphistomatic	1	1	1	1	1
5	Stomata On adaxial	Low frequency	1	0	1	1	1
		High frequency	0	1	0	0	0
6	Predominant stomata (Ps)	Ani	1	0	1	0	0
		Ani/Para/Bp	0	1	0	0	0
		Para/Bp	0	0	0	0	1
		Para	0	0	0	1	0
7	Glandular trichomes (Gt)	Present	1	1	1	1	1
8	Simple falcate trichomes (F)	Predominant	0	1	0	0	0
		Intermixed	1	0	1	1	1
9	Conical (Cn)	Intermixed and Predominant	0	0	0	0	1
		Intermixed	1	0	1	1	0
10	Bi-furcate and stellate (Bi & S)	Present	1	0	1	1	1
		Absent	0	1	0	0	0
11	Petiole outline/ Middle (P)	Semi-terete	1	1	1	1	1
12	Adaxial groove (Ag)	Straight flattened	0	0	0	1	1
		Shallow grooved	1	0	1	0	0
		Grooved	0	1	0	0	0
13	Crystal	Druses	1	1	1	1	1
14	Main vasculature (Mv)	Three in number	1	1	1	1	1
15	Accessory vascular bundles (Ac)	One	0	0	0	1	1
		Three-four	1	0	1	0	0
		Six-eight	0	1	0	0	0
16	Epidermal secretory idioblasts (Ei)	Present	1	1	1	1	1
17	Secretory ducts (Sd)	Present	0	1	0	1	1
		Absent	1	0	1	0	0
18	Mucilage idioblasts (Mi)	Present	1	1	1	1	1
19	Mucilage idioblasts size (Mi)	Large-sized	0	0	0	0	1
		Medium-sized	1	1	1	1	0
20	Mid rib outline	Biconvex	1	1	1	1	0
		Flat-convex	0	0	0	0	1
21	Sf	Present	1	1	1	1	1

Ap: Alternate percurrent, **Ani**: Anisocytic, **Ba**: Basal actinodromous, **Bp**: Brachyparacytic, **C**: Craspedodromous, **Fb**: Festooned brochidodromous, **Fsc**: Festooned semi-craspedodromous, **M/opp/alt**: Mixed opposite alternate percurrent, **Para**: Paracytic, **Sf**: Sclerenchyma fibers in leaf, **(+)**: detected, **(-)**: not detected.

Table S2 Data matrix of GC-MS phytochemical compounds applied in numerical analysis

No	Compound Name	Area %				
		H. rosa sinensis	*H. sabdariffa*	*H. schizopetalus*	*H. syriacus*	*H. tiliaceus*
1	d-Glycero-d-galacto-heptose	**0**	1	1	**0**	**0**
2	L-Glucose	**0**	**0**	**1**	**0**	**0**
3	14-Pentadecenoic acid	**0**	**0**	**1**	**0**	**0**
4	Xanthinin	**0**	**0**	**1**	**0**	**0**
5	α-Methylionol	**0**	**0**	**1**	**0**	**0**
6	β-Hydroxydodecanoic acid	**0**	**0**	**1**	**0**	**0**
7	α,β-Gluco-octonic acid lactone	**0**	**0**	**1**	**0**	**0**
8	3',4',7-Trimethylquercetin	**1**	**1**	**1**	**1**	1
9	Phytol	**1**	**1**	**1**	**1**	1
10	3-Methylkaempferol	**1**	**1**	**1**	**1**	1
11	10-Octadecenal	**1**	**1**	**1**	**1**	1
12	cis-10-Nonadecenoic acid	**0**	**0**	**1**	**0**	**0**
13	Methyl palmitate	**1**	**1**	**1**	**1**	1
14	Oleic Acid	**1**	**1**	**1**	**1**	1
15	Palmitic acid	**1**	**1**	**1**	**1**	1
16	Flavonol 3',4',5,7-OH,3-O-araglucoside	1	**0**	1	**0**	**1**
17	Squalene	**1**	**1**	**1**	**1**	1
18	Isolongifolol	**1**	**1**	**1**	**1**	1
19	(-)-Citronellol	**1**	**1**	**1**	**1**	1
20	Linoleic acid	**1**	**1**	**1**	**1**	1
21	(S)-(-)-Citronellic acid	**1**	**1**	**1**	**1**	1
22	Retinoic acid	**1**	**1**	**0**	**1**	1
23	18α-Glycyrrhetinic acid	**1**	**1**	**1**	**1**	1
24	DELTA.9-Tetrahydrocannabinol	**1**	**0**	**1**	**1**	1
25	Cannabidiolic acid	**1**	**0**	**0**	**1**	1
26	trans-Trismethoxyresveratrol	**0**	**0**	**1**	**0**	**0**
27	Apigenin 8-C-glucoside	**1**	**0**	**1**	**1**	1
28	Pregnan-20-one	**1**	**1**	**1**	**1**	1
29	α-Tocopherol	**1**	**1**	**0**	**0**	**0**
30	β-Guaiene	**0**	**0**	**1**	**0**	**0**
31	Arachidonic acid methyl ester	**0**	**0**	**1**	**0**	**0**
32	β-Sitosterol	**1**	**1**	**0**	**1**	1
33	Isopatchoulenone	**0**	**0**	**1**	**0**	**0**
34	Campesterol	**1**	**0**	**1**	**0**	**1**
35	Spinacene	**1**	**0**	1	**1**	1
36	Stigmasterol	**1**	**1**	**0**	**1**	1
37	(-)-Catechin gallate	**1**	**1**	**1**	**1**	1
38	5,7,4'-Trimethoxyisoflavone	**1**	**1**	**1**	**1**	1
39	Cyanin cation	**1**	**1**	**0**	**0**	**1**
40	Isoorientin	**0**	**0**	1	**0**	**1**

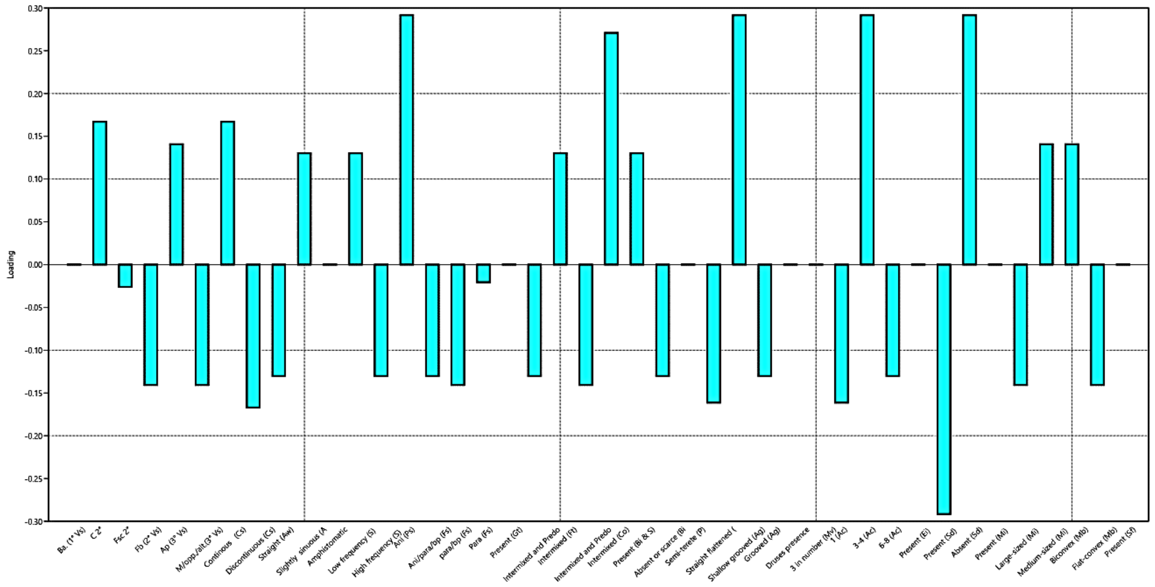

Fig. S1 The loading scores of each variable (42 character states, Table S1) from traditional morphoanatomical characters on the most variable principal component (PC1).

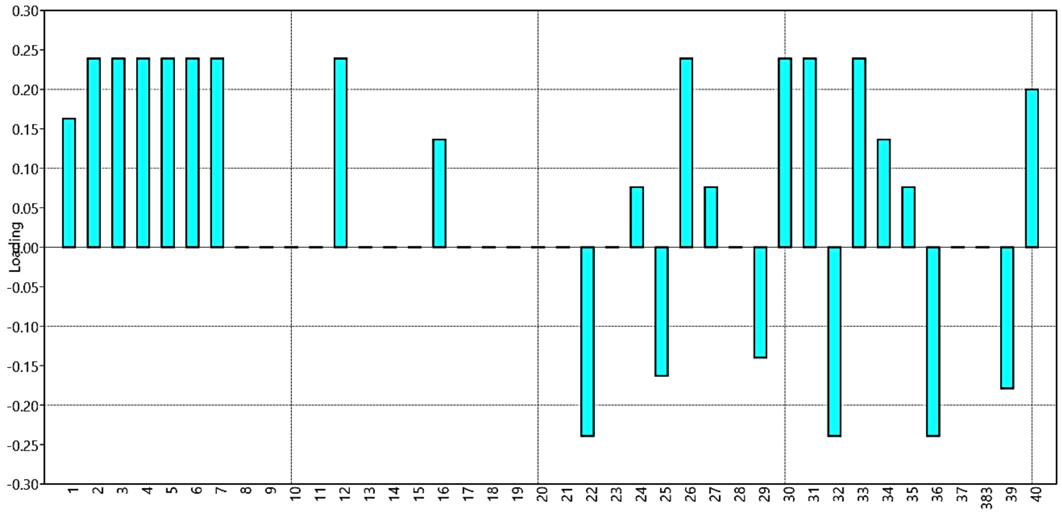

Fig. S2 The loading scores of each variable (40 phytochemical compounds, shown in Table S2) from GC-MS analysis of the methanolic leaf extract using the most variable principal component (PC1).
